# 肺移植术后闭塞性细支气管炎综合征预测标记物研究进展

**DOI:** 10.3779/j.issn.1009-3419.2020.101.03

**Published:** 2020-06-20

**Authors:** 思佳 杨, 阿布都买拉木·阿布都吾甫尔, 望 吕, 飞潮 包, 坚 胡

**Affiliations:** 310003 杭州，浙江大学医学院附属第一医院 The First Affiliated Hospital, Collage of Medicine, Zhejiang University, Hangzhou 310003, China

**Keywords:** 肺移植, 闭塞性细支气管炎综合症, 预测标记物, Lung transplantation, Bronchiolitis obliterans syndrome, Predictor

## Abstract

肺移植是治疗终末期肺病的有效方法。目前，肺移植术后1年生存率已达到80%，由于闭塞性细支气管炎综合症（bronchiolitis obliterans syndrome, BOS）的发生，5年生存率维持在50%左右。BOS是一个纤维化的过程，最终导致不可逆的气道闭塞。缺血-再灌注损伤、感染、氧化应激以及急性排斥反应等多个因素参与了BOS的发生。研究证实BOS的早期诊断与预后良好相关。因此，寻找灵敏、特异的BOS预测标记物对于提高肺移植患者长期生存具有重要的科学和临床意义。本文就与BOS发生发展相关的免疫调节细胞、分泌性蛋白质、细胞膜蛋白等指标的变化在BOS早期诊断中的作用进行综述。

## Abstract (en)

Lung transplantation is the ultimate treatment of end-stage lung disease. After transplantation, the 1-year survival rate is 80%, while the 5-year survival rates remaines at around 50% mainly due to bronchiolitis obliterans syndrome (BOS). BOS is regarded as a fibrosing process in the small airways leading to irreversible airway obstruction. A lot of factors are involved in the development of BOS, such as Ischemia/reperfusion injury, infections, oxidative stress, and acute rejection, etc. Studies have shown that early diagnosis of BOS may improve outcome. It is valuable for the long-term survival of lung transplantation to find out several predictors for the BOS. This article reviews the current state of knowledge on predictors for BOS.

肺移植是治疗慢性阻塞性肺疾病、特发性肺纤维化、肺囊性纤维化等终末期肺部疾病的有效方法^[1]^。但是，肺移植患者的长期生存率显著低于其他实体器官移植患者^[2]^。慢性移植物功能障碍是限制肺移植患者长期生存的主要原因^[3]^，由于术后闭塞性支气管炎（bronchiolitis obliterans, BO）等并发症的发生，肺移植患者5年生存率约为50%，而10年生存率仅为30%^[4]^。BO病理表现为间质细胞浸润终末呼吸道并引起纤维增生，闭塞性细支气管炎综合症（bronchiolitis obliterans syndrome, BOS）是BO的临床表述，表现为肺功能进行性不可逆的下降，并排除急性排斥反应，感染或者吻合口并发症等原因^[5]^。目前，BOS主要的治疗策略是抗炎治疗，缺乏特异性的抗纤维化治疗手段^[6]^。早期BOS患者可通过干预性治疗延缓BOS的病程进展，因此寻找具有良好相关性、且诊断敏感性较高的指标，有利于BOS的早期诊断和治疗。近年来研究证实，BOS发生发展相关的免疫调节细胞、细胞因子、金属蛋白酶及抑制剂等指标具有不等程度的作为BOS早期诊断标记物的价值。本文就一部分相关研究较多、预测价值相对高的支气管肺泡灌洗液（bronchoalveolar lavage fluid, BALF）和外周血中的生物标记物研究进展进行综述。

## 1 中性粒细胞（neutrophilia, NE）

NE中性粒细胞在BOS的发生发展过程中发挥着重要作用。气管内NE增多导致成纤维细胞增殖和抗氧化防御枯竭，进而造成气道重塑和纤维化^[7]^。Neurohr等^[8]^报道了一项前瞻性研究，检测63例肺移植患者术后3个月-12个月BALF中NE的含量；当NE比例≥20%时，预测BOS的灵敏性为72%，特异性为93%。Verleden等^[9]^对380例肺移植患者随访发现，发展成BOS的79例患者术后第6、12、18和24个月BALF中NE含量均显著高于正常肺移植患者（*P* < 0.05, *P* < 0.01, *P* < 0.001, *P* < 0.000, 1）。Berastegui等^[10]^研究证实BOS组的BALF中，NE中位数和INF-γ水平显著高于稳定组，BALFNE可作为预测BOS的标志。目前，已有较多报道^[11-15]^表明BALF中NE比例可以早期预测BOS的发生。在排除急性排斥反应或淋巴细胞性支气管炎等影响NE含量的干扰因素后，BALF中NE比例升高在预测BOS中的价值已被认可。

## 2 调节性T细胞

调节性T细胞通过抑制效应性CD4^+^ T细胞和CD8^+^ T细胞的过度活化，在维持移植耐受和控制自身免疫反应中具有重要作用，其含量的异常与BOS的发生密切相关^[16]^。Bhorade等^[17]^评估了测定调节性T细胞（T-lymphocytes regulatory, Treg）含量在预测BOS中的价值，通过流式细胞仪比较了14例正常肺移植患者和6例发展为BOS的肺移植患者血液和BALF中T细胞FoxP3的表达水平，发现正常肺移植患者BALF中FoxP3^+^细胞在CD4^+^ T细胞中所占的比例高于发展成为BOS的肺移植患者；同时发现术后2年内，BALF中CD4^+^FoxP3^+^ T细胞含量3.2%可作为预测BOS的临界值，而且，FoxP3在CD4^+^ T细胞中的比例不仅可以预测BOS，还能指导肺移植患者免疫抑制治疗。Nakagiri^[18]^通过随访18例肺移植患者，提出了类似的观点，认为调节性T细胞升高预示着患者术后肺功能良好。Durand等^[19]^发现，肺移植术后1个月，外周血中CD4^+^CD25^hi^FoxP3^+^ T细胞含量2.4%可作为区分BOS患者和稳定患者的界限，移植后6个月CD4^+^CD25^hi^FoxP3^+^ T细胞含量2.4%以上的患者患BOS的风险是其他患者的2倍。但也有学者持不同意见，Krustrup等^[20]^认为组织活检中FoxP3^+^细胞的密度不能作为预测BOS的指标。

## 3 CD4^+^CD28^+^ T细胞

CD28作为协同刺激分子，与B7家族分子结合产生T细胞活化的共刺激信号在免疫应答的T细胞激活中起重要作用，CD28表达异常与变态反应、慢性免疫疾病、移植耐受等疾病有关。Studer等^[21]^采用流式细胞术分析肺移植患者外周血CD4^+^ T细胞CD28的表达情况，移植术后6个月内，CD4^+^CD28^+^/CD4^+^ T细胞比例低于90%的患者第1秒用力呼气量（forced expiratory volume in the first second, FEV_1_）下降（0.35±0.04）L，而CD4^+^CD28^+^/CD4^+^ T细胞比例高于90%的患者FEV_1_下降（0.08±0.08）L，两组间有显著性差异（*P* < 0.013）；且CD4^+^CD28^+^/CD4^+^ T细胞比例≤90%时，预测BOS发生的特异性为88%。Hodge等^[22]^也得出了相似的结果；通过分析58例肺移植患者外周血中CD4^+^ T细胞和CD8^+^ T细胞表达CD28的情况，他们发现正常肺移植患者与发展成BOS的患者外周血中淋巴细胞总数及CD4、CD8在T细胞中百分比无显著差异，而CD4^+^CD28^-^/CD4^+^ T细胞、CD8^+^CD28^-^/CD8^+^ T细胞比例有显著差异，并且CD8^+^CD28^-^/CD8^+^ T与FEV1相关（*r*=-0.675, *P*=0.001）。上述研究证实CD4^+^ T细胞和CD8^+^T细胞低表达CD28可提示移植后较差的肺功能和BOS的发生，对BOS的发生起到预测作用。

## 4 间充质干细胞

间充质干细胞（mesenchymal stromal cells, MSC）是纤维增殖的初始效应细胞，在组织器官动态平衡、损伤后修复的过程中发挥重要作用^[23]^。Badri等^[24]^测定162例肺移植患者术后不同时间点BALF中MSC菌落形成单位（colony-forming units, CFU）的数量，发展成BOS的患者BALF中间充质干细胞CFU数量显著高于正常肺移植患者；研究结果表明移植术后6个月，BALF中间充质干细胞CFU≥10可以预测BOS的发生。MSC在肺部疾病的作用机制尚不清楚，有研究^[25]^表明MSC是通过内皮素（endothelin-1, ET-1）发挥其促BOS的作用。目前，对于MSC达到多少数量才可以早期预测BOS的发生，以及MSC数量是否与BOS的程度相关尚未定论。

## 5 CC趋化因子

CC趋化因子具有募集淋巴细胞、单核细胞、粒细胞的作用，目前CC趋化因子在BOS病理过程中的重要作用已被重视^[26]^。研究^[27]^提示肺移植术后血清中胸腺和活化调节趋化因子（CCL17/TARC）水平可以作为BOS的预测指标。Paantjens等^[24]^测定了54例肺移植患者术前、术后血清中TARC水平，发现测定术后第1年内血清TARC水平有助于预测BOS，TARC≤325 pg/mL的患者发展成为BOS的特异性和灵敏性分别为71%和80%。Reynaud等^[28]^建议将其连同另一个CC趋化因子MCP-1作为预测BOS早期敏感诊断的指标。Verleden等^[29]^通过检测77例肺移植患者BALF中34种不同细胞因子的表达情况，发现正常肺移植患者与发展成BOS的患者BALF中MCP-1、CCL3、CCL4、CCL17、CCL18的表达有显著差异。上述研究提示检测CC趋化因子对预测BOS有一定的价值。

## 6 可溶性蛋白

T细胞介导的自身免疫反应在BOS的发生中具有重要的作用^[30]^。当CD30^+^的T细胞活化后，释放可溶性CD30（solubleCD30、sCD30）入血。Bauwens等^[31]^在术前检测38例肺移植患者血清中sCD30的水平，发现术前高水平sCD30（> 20 U/mL）的患者与低水平sCD30（≤20 U/mL）患者相比有显著的无BOS生存期优势。Golocheikine等^[32]^通过连续测定肺移植患者血清sCD30水平，发现发展成为BOS的患者较未发展成为BOS的患者血清sCD30升高（*P* < 0.05），在临床诊断BOS的（7.57±2.63）个月前就发现了此差异，因此其建议将血清sCD30升高作为BOS的早期预测指标。Fields等^[33]^持续跟踪40例肺移植患者血清sCD30水平得到与Golocheikine相同的结论。

Kwakkel等^[34]^认为不能将sCD30作为BOS的预测指标，他们测定了14例接受他克莫司/霉酚酸酯治疗方案的肺移植患者血清样本中的sCD30水平，其中7例患者发展成为BOS，但在移植术前和术后均未发现与正常患者相比sCD30有统计学差异。Kwakkel的研究证明了他克莫司/霉酚酸酯不能通过抑制sCD30阻止BOS的发生，但未对sCD30在BOS预测中的价值进行直接反驳。sCD30作为CD30活化释放入血的活性成分，与CD30具有很好的相关性。血清中sCD30水平能够反映活化的CD30^+^细胞数量。因此，测定血清sCD30水平在BOS预测中有一定的价值。

除可溶性CD30外，可溶性补体蛋白CD59（sCD59）也可作为BOS的预测指标。Budding等^[35]^检测了肺移植后BOS患者和非BOS患者的血清sCD59含量，发现sCD59量与BOS显著相关。sCD59滴定 > 400 pg/mL时，术后6个月患者生存率显著低于sCD59滴定低组。

## 7 KL-6

KL-6表达于Ⅱ型肺泡上皮细胞及呼吸性细支气管上皮细胞，具有募集纤维母细胞促进肺间质纤维化的作用，血清KL-6水平与肺泡损伤、Ⅱ型肺泡细胞再生和多种间质性肺疾病的严重程度有关^[36]^。受损伤的肺泡上皮细胞释放KL-6促进成纤维细胞分化成肌成纤维细胞^[37]^。Walter等^[38]^检测了26例肺移植患者血清KL-6水平，其中8例为BOS患者，发现血清KL-6水平同FEV_1_下降相关（*r*=0.44, *P* < 0.05），BOS患者与正常肺移植患者血清KL-6浓度的平均数±标准偏差分别为（688.7±225.8）U/mL、（321.3±163.9）U/mL，该结果说明血清KL-6检测有作为BOS早期诊断指标的潜力。最近Haberman等^[39]^的研究也证明了血清KL-6在诊断BOS中的价值，KL-6≥200 U/mL早期预测BOS的敏感性为67%，特异性为95%；因此认为血清KL-6可以作为移植术后早期诊断BOS的特异性指标。Ohshimo等^[40]^比较了BALF中NE和血清KL-6对BOS的预测价值，认为检测血清KL-6较BALF中NE更有助于早期诊断BOS。

## 8 Clara细胞分泌蛋白

Clara细胞分泌蛋白（Clara cell secretory protein, CCSP）由分布于终末细支气管和呼吸性细支气管的无纤毛上皮细胞（Clara细胞）分泌，具有抗炎、抗纤维化的生物学活性。上皮损伤或非正常修复后，再生的上皮细胞失去分泌CCSP的能力。Nord等^[41]^进行了一项包含22例肺移植患者的队列研究，发展成为BOS的患者与急性排斥反应或者无排斥反应的患者相比，其血清和BALF中CCSP显著下降，表明检测血清和BALF中CCSP水平可以预测BOS的发生。Bourdin等^[42]^随访63例肺移植患者，发展成为BOS的患者在移植术后3个月内BALF中CCSP水平和CCSP/IL-8比率已低于正常患者，并持续性下降，验证了BALF中CCSP下降对于BOS的预测作用，同时提出供体*CCSPA38G*基因多态性与患者术后并发BOS相关。上述研究均表明术后CCSP下降，尤其是BALF中CCSP下降对BOS的预测作用。因此，CCSP的表达可能对BOS的发生有一定的预测作用。

## 9 内皮素1

内皮素1（endothelin-1, ET-1）是由21个氨基酸组成的多肽，具有调节血管舒缩张力、激素分泌等功能^[43]^。研究^[43, 44]^表明ET-1对肿瘤，自身免疫疾病等的发生发展有影响。ET-1在肺移植物失功中的病理生理过程中发挥着重要作用，对预测移植物失功也有一定作用^[25]^。Salama等^[45, 46]^证实了ET-1浓度升高在BOS预测中的价值，ET-1主要通过控制间充质细胞来促进BOS的发生；*Logistic*回归分析发现，术前ET-1浓度和术后3个月、12个月的血清ET-1浓度升高可以预测BOS，单因素比值比（univariate OR）和95%置信区间（confidence interval, CI）分别为1.01（1.004-1.025）、3.1（1.21-9.92）、3.9（1.42-10.80）。上述研究说明ET-1在BOS的发生发展中起着重要作用，有可能通过控制其他细胞促纤维增生而使终末呼吸道纤维化，同时检测血清和BALF中ET-1浓度在一定程度上可以预测或早期诊断BOS的发生。

## 10 金属蛋白酶（matrix metalloproteinases, MMPs）和金属蛋白酶组织抑制因子（tissue inhibitors of metalloproteinases, TIMPs）

上皮细胞损伤、炎症、纤维化及气道闭塞与术后闭塞性细支气管炎相关^[47]^，纤维化是纤维增生（即细胞外基质合成增加）和纤维分解（即细胞外基质降解）不平衡的结果，包括MMPs和TIMPs在内的蛋白质参与胞外基质成分平衡的维持^[48]^。研究^[49]^证实，MMPs和TIMPs在BOS患者肺组织中有表达改变。Hubner等^[50]^对出现肺纤维增生影像学表现的肺移植患者跟踪测定MMP-9和TIMP-1的表达，结果显示，BOS患者的移植肺表达MMP-9和MMP-9/TIMP-1比值均显著升高。Smith等^[51]^研究证实BALF中MMP-8、MMP-9和TIMP-1在术后2年内升高与之后发生BOS相关。亦有报道^[12, 51-58]^表明MMPs家族的其他成员如MMP-1、MMP-2、MMP-3和MMP-12等表达的改变与BOS发生相关，但这些MMPs在预测BOS中的价值现证据不够充分。

## 11 MiRNAs和基因表达

Xu等^[58]^检测了肺移植后儿童体内循环miRNA的水平，发现BOS患者的miR-134、miR-10a、miR-195和miR-133b显著低于非患病组，而miR-144、miR-142-5p和miR-155显著低于非患病组。Budding等^[59]^认为BOS患者血清中miR-21、miR-29a、miR-103和miR-191的水平显著高于非BOS组。提示特定的miRNAs可作为预测BOS的指标。

此外，Danger等^[60]^利用qPCR检测外周血中的基因表达，对比BOS组和非BOS组，提出*CD19*、*BLK*、*POU2AF1*、*TCL1A*和*IGLL5*五种可作为预测标记的高表达基因，以及*IGLL5*和*TCL1A*两种可作为诊断标记的基因。

## 12 结语

肺移植是治疗终末期肺病的有效方法。受制于移植术后BOS的发生，肺移植长期生存率较低^[1]^，BOS的中位生存时间仅为3年-4年。寻找BOS的预测指标，提高BOS的早期发现率并提前干预，有助于延长肺移植患者的生存^[6]^。经过多年研究，已经证实免疫调节细胞、分泌性蛋白质等指标在预测肺移植术后BOS的发生中具有一定的价值，如NE、Treg、CD4^+^CD28^+^ T细胞、白介素、C反应蛋白、ET-1、KL-6等；其中NE、sCD30对预测BOS的研究较多，临床应用的价值较为认可。同时，也有学者在探索其他类型的标记物，如转化生长因子-β（transforming growth factor-β, TGF-β）^[62]^、微生物^[63]^、呼气一氧化碳^[64]^、YKL-40^[65]^、1H NMR^[66]^、HLA-G^[67]^等。随着BOS发生机制研究的深入，大样本临床试验的开展，更多高灵敏度、特异性的BOS预测指标将为肺移植术后BOS患者带来长期生存的希望。
